# New York Letter

**Published:** 1890-11

**Authors:** 


### Correspondence.

### NEW YORK LETTER.

NEW YORK, OCT, 17, 1890.

The winter season in this city has now fully opened and the arrival of large numbers of medical men from both the states and Canadas has given a fresh stimulus to medical matters. These physicians come here to take a post-graduate course and no city in the world offers a more fertile field for that purpose than does New York. It may be safely said that over half a million sick people are being treated gratuitously each year in its numerous hospitals and dispensaries, and this, too, despite the fact that there are from eight to ten thousand regular physicians in this city, who are dependent for their support wholly on the tenement house districts, which supply this vast army of invalids.

It is both an interesting and instructive lesson to visit the clinics of some of the well-known physicians of this city and see the motley array of human beings applying for relief. All the various ills that human flesh is heir to are there typified, and he would indeed be wholly indifferent to medical progress, who would depart not highly instructed.

At a recent clinic at the University Medical College, Prof. Wm. H. Thomson, speaking of functional and organic nervous diseases, laid down the following dictum: True functional disorders can have no common origin with the organic nervous diseases, as no organic nervous disease exists whose symptoms are intermittent.

True functional nervous diseases are always peripheric in their origin and never centric.

He also stated that in every case of epilepsy he always examines very carefully into the possible sources of apparent irritation. He has repeatedly found the first indication of the kind in the alimentary canal, and by perseveringly dealing with that organ, he has finally succeeded in getting a mastery of the malady. As an illustration of that he gave the following instance: A young man, eighteen years of age, had been brought to him, who had fits ever since he was six years of age, and had been treated by various physicians, but without success. His attacks averaged twelve a week and did not yield in the least to bromide. He was treated by him for two months, but without any material benefit, until the parents informed him that just preceeding or immediately following an attack the boy had a diarrhœa. He was ordered half a grain of picrate of ammonium as an intestial disinfectant four times a day. The fits immediately ceased and he has had but one attack since in eleven months.

When irritation within the cranium can be inferred from twitching of the muscles of the face or extremities during sleep, he generally added corrosive sublimate or the red iodide of mercury to the bromides, when these are well borne, and has found the bromides then succeed when they had previously failed. He explained the action of the bromides in epilepsy from the fact of their being such powerful sedatives to reflex actions.

In the treatment of hysteria he has found nothing so positive in its effect as a purge with half an ounce of turpentine every few days, followed by persistent employment of intestinal antiseptics.

Prof. Fordyce Barker, of this city, does not think that the existence of albuminuria in pregnant women, even in its greatest development, unless accompanied with undoubted evidences of perilous uræmia, and unless other resources for the cure of both the albuminuria and the uræmia have been exhausted, justifies the induction of premature labor. At a recent clinic held in the amphitheatre of Bellevue Hospital, he gave it as his conviction that by appropriate prophylactic treatment the albuminuria and uræmia may be absolutely averted. He has not had in years a case of eclampsia in a patient of his who had been treated in that way. He spoke of one agent in the treatment of the uræmia of the pregnancy, which will prove of great value—nitro-glycerine, in doses of from 1-100 to 1-50 of a minim every three or four hours. Its effect, he stated, was to speedily reduce arterial tension and allay spasm of the cerebral and renal arterioles, and thus indirectly increase the functional activity of the kidney and quiet the nerve storm, so characteristic in these patients.

The question of diet, he considers of the greatest importance in this class of patients. He divided these patients into two classes, one in whom anemia, hydræmia and general feebleness of the vital powers predominated, and in the other plethora and abnormal activity of the digestive and assimilative functions.

The first class need a nutritious diet, avoiding the danger of overtaxing the kidneys by restricting the amount of nitrogen. He has seen great benefit in giving these patients, weeks before parturition, a teaspoonful of a mixture of glycerine, three parts, and tincture of the chloride of iron, one part, in a wine glass of water, after each meal.

In the other class of cases, he recommends an exclusive milk diet. He questions the utility of diuretics in these cases, except the indirect diuresis, which results from digitalis and the tincture of the chloride of iron. If the albuminuria is attended with symptoms threatening uræmia, or uræmia already exists, then more active treatment is necessary. He regards venesection as of the first importance. The bleeding, he says, removes tension from the brain, relieves congestion of the lungs, makes the breathing freer and eases the congested kidney of its burden.

Speaking of the etiology of this affection, he believe albuminuria to be due to certain modifications of the system resulting from changes in the quality of the blood. During the period of gestation the blood becomes super-albuminous. There is increased demand for albumin for the nourishment of the fœtus. Therefore, the maternal albumin, which passes through the fœtus without being employed in its growth, returns to the maternal system loaded with waste material.

We have at present writing an epidemic here—not of scarlet fever, measles or diphtheria, but of a worse variety than either—an epidemic of the blatant and irrepressible quack. This organization may be said to owe its existence here wholly to the greed and rapacity of the daily press, though its etiology at the present juncture is buried in obscurity. The ignorant and credulous, the unmitigated mean, and the undiluted crank, fall easy victims to seductive pictorial advertisements of these creatures, and the story of the spider and the fly is again retold with great suggestiveness. The writer was recently told by a reputable druggist of this city that he has received and filled out prescriptions from well-known regular physicians recommending to their patients the use of a certain patent nostrum, prepared by the most notorious quack of New York. If this be true—and there is no reason to doubt its accuracy—it is a most fearful reflection on the intelligence of some of our professional brethren in this city, and goes in a great measure to explain the cause of this present invasion.

Dr. Alfred L. Loomis, speaking at a recent clinic on the subject of cardiac dilatation, said the most promising variety of cardiac dilatation is that depending on general impaired nutrition, associated with extreme anemia. The most common example of this variety is met with in poor working women, who live exclusively on bread and tea. It is of the utmost importance that these patients should have healthful surroundings, and they should spend at least eight hours of the day in the open air. Their diet must be nutritious and non-stimulating and include a large proportion of animal food. The only remedial agent he has found of service was arsenic in 1-60 grain doses, three or four times a day after meals· This plan, if rigidly enforced, will cause all signs of cardiac dilatation to disappear, and the œdema of the lower extremities, so constantly present, will rapidly subside.

In dilatation due to softening of the cardiac muscle, so often present during convalescence from typhoid fever and other acute infectious diseases, he recommends keeping the patients in bed and not allowing them, under any circumstances, to assume the upright position for at least four weeks. They should be given a large amount of liquid nourishment and a free allowance of wine. Everything tending to excite the circulation should be avoided, as well as those influences which dispose to cardiac enfeeblement. The medicinal agent he recommends is 2 grains of quinine and 1-60 of a grain of strychnine three times a day.

For dilatation due to fatty degeneration, he advises the use of small doses of calomel combined with rhubarb and soda, a spare diet and absolute rest. This will do more, he says, to equalize the circulation and relieve the distressing symptoms than any tonic measures addressed to the heart itself. In emergencies, when the dyspnœa is urgent and the heart's action irregular and intermittent, a full hypodermic of morphia may be given.

For dilatation due to fibroid degeneration, bichloride of mercury should be administered in 1-60 grain doses, three times a day for a long period, and all the so-called heart tonics are to be avoided.

For dilatation due to excessive use of tobacco or narcotics, he advises the gradual withdrawal of the cause and the administration of full doses of strychnine, with the daily use of cold shower baths, night and morning; and for cardiac dilatation resulting from venery, he recommends the use of thirty grains of bromide of potash, three or four times a day for several weeks. P. J. R.

---

ANTIDOTE TO CHLOROFORM AND COCAINE..—Nitrite of amyl is commended as the most rational and successful antidote to use where chloroform or cocaine seems to threaten life by their unfavorable action on the heart. A few drops of nitrite of amyl administered by inhalation, will be one of the most probable means of restoring the heart's action.—*Journal American Medical Association.*

---

COFFEE A SLOW POISON.—A physician told Fontanelle that coffee was a poison. "Doctor," said the Acadamician, "I believe, like you, that coffee is a slow poison, for I have taken it for eighty years."

